# An in-depth assessment of a diagnosis-based risk adjustment model based on national health insurance claims: the application of the Johns Hopkins Adjusted Clinical Group case-mix system in Taiwan

**DOI:** 10.1186/1741-7015-8-7

**Published:** 2010-01-18

**Authors:** Hsien-Yen Chang, Jonathan P Weiner

**Affiliations:** 1 Department of Health Policy & Management, Bloomberg School of Public Health, Johns Hopkins University, 624 N Broadway St, Baltimore, MD 21205, USA

## Abstract

**Background:**

Diagnosis-based risk adjustment is becoming an important issue globally as a result of its implications for payment, high-risk predictive modelling and provider performance assessment. The Taiwanese National Health Insurance (NHI) programme provides universal coverage and maintains a single national computerized claims database, which enables the application of diagnosis-based risk adjustment. However, research regarding risk adjustment is limited. This study aims to examine the performance of the Adjusted Clinical Group (ACG) case-mix system using claims-based diagnosis information from the Taiwanese NHI programme.

**Methods:**

A random sample of NHI enrollees was selected. Those continuously enrolled in 2002 were included for concurrent analyses (*n *= 173,234), while those in both 2002 and 2003 were included for prospective analyses (*n *= 164,562). Health status measures derived from 2002 diagnoses were used to explain the 2002 and 2003 health expenditure. A multivariate linear regression model was adopted after comparing the performance of seven different statistical models. Split-validation was performed in order to avoid overfitting. The performance measures were adjusted R^2 ^and mean absolute prediction error of five types of expenditure at individual level, and predictive ratio of total expenditure at group level.

**Results:**

The more comprehensive models performed better when used for explaining resource utilization. Adjusted R^2 ^of total expenditure in concurrent/prospective analyses were 4.2%/4.4% in the demographic model, 15%/10% in the ACGs or ADGs (Aggregated Diagnosis Group) model, and 40%/22% in the models containing EDCs (Expanded Diagnosis Cluster). When predicting expenditure for groups based on expenditure quintiles, all models underpredicted the highest expenditure group and overpredicted the four other groups. For groups based on morbidity burden, the ACGs model had the best performance overall.

**Conclusions:**

Given the widespread availability of claims data and the superior explanatory power of claims-based risk adjustment models over demographics-only models, Taiwan's government should consider using claims-based models for policy-relevant applications. The performance of the ACG case-mix system in Taiwan was comparable to that found in other countries. This suggested that the ACG system could be applied to Taiwan's NHI even though it was originally developed in the USA. Many of the findings in this paper are likely to be relevant to other diagnosis-based risk adjustment methodologies.

## Background

Risk adjustment has become an increasingly important tool in healthcare around the globe. It is being extensively applied to provider performance assessment [1-3], high risk predictive modelling for disease management [4-6] and payment adjustment [7-10]. Through these applications the broader goals of equity, efficiency and improved outcomes may be achieved in a healthcare system. Health indicators that are often used for risk adjustment include demographic factors, subjective/self-reported health status [11,12], biomedical clinical indicators [13,14], prior expenditure [15,16] and claims-based morbidity burden indicators (using diagnostic codes [17,18] or medication codes [9,19-21]). Even though demographic factors are most often used given the availability of the data, both prior-expenditure and claims-based health indicators perform much better than demographic factors [8,9,13,14,16,19,22]. However, since risk adjustment models are usually adopted for payment adjustment, models using diagnosis and/or pharmacy data are preferred as prior use models could offer inappropriate incentives to increase services in order to receive higher payments. Diagnosis and/or pharmacy-based risk adjustment models have been developed and gradually adopted in Canada, the USA and Europe [9,23-29].

The Johns Hopkins Adjusted Clinical Group (ACG) system is a comprehensive risk adjustment software that incorporates diagnosis information, pharmacy information, or both, to capture an individual's morbidity burden [23,24,30]; ACG actuarial cells and Aggregated Diagnosis Group (ADG) binary morbidity markers have been extensively examined in the USA [8,16], Canada [31] and other European countries [32]. However, Expanded Diagnosis Clusters (EDCs), another output of the ACG system, have not been assessed carefully in terms of performance on risk adjustment. EDCs are binary indicators which show whether or not an individual has specific diseases/symptoms. It has been shown that a substantial fraction of health costs resulted from the treatment of a relatively small number of common, but expensive, chronic diseases [15]. Therefore, our supposition is that the basic ACG models can be improved upon by the inclusion of selected disease conditions (represented by EDCs) [17].

Taiwan launched a government-run, single-payer National Health Insurance (NHI) programme in May 1995. All Taiwanese nationals are obligated by law to join this programme to ensure adequate risk pooling. Under the jurisdiction of the national government's Department of Health, the NHI is administered by the Bureau of National Health Insurance (BNHI) and six regional branches are in charge of administrating the NHI in each area. The NHI's benefit packages are comprehensive, including inpatient and outpatient services, pharmacy services, Chinese medicine and dental services. Beneficiaries have complete freedom of choice of providers and therapies, and they do not need to go through 'gatekeepers' in order to obtain medical services from specialists. The primary source of funding for the NHI is the payment of premiums shared by the insured, the employers and the government. In terms of reimbursement, the global budget payment system was adopted in order to contain the growth of medical expenditure. Within budget limits, the NHI reimburses contracted providers mostly on a fee-for-service basis, using uniform national fee schedules. Entering the second decade, reform of Taiwan's NHI has focused on three aspects: quality improvement; financial balance; and expansion of social participation. In order to achieve the first two goals, the implementation of risk adjustment is crucial [33].

Diagnosis-based risk adjustment is still a very new concept in Asia and all existing risk adjustment technologies were developed using claims data from Western countries. Several studies evaluated their performance in Taiwan [34,35]. However, they were either methodologically limited (for example, split-validation was not performed), only reported a single measure at individual level (R^2^), or simply focused on total expenditure. Given that diagnosis-based risk adjustment has different implications at individual and group level (such as budget allocation) and across different types of expenditures, it is necessary to thoroughly examine risk adjustment models before they can be directly applied in Taiwan. In addition, in most cases, previous evaluations of risk adjustment models have used regional datasets or focused only on sub-population. Taiwan is one of very few health care systems in the world which has universal coverage and a single national computerized database that includes medical diagnosis information on almost 100% of the population. For this reason the results of this paper have potential policy and methodology implications for most other high or middle income nations.

In this paper we aimed to assess the performance of the ACG system using Taiwan's NHI claims data and to evaluate how adding EDCs could affect the performance of the ACG system.

## Methods

### Data sources

The source of the data was a longitudinal dataset prepared by Taiwan's BNHI, which is available for researchers interested in observing longitudinal changes of medical utilization. Individuals' identifiers in this dataset have been encrypted in order to protect privacy and confidentiality, and this study has been approved by the Johns Hopkins School of Public Health Institutional Review Board. This dataset contained enrollment and claims files of a randomly chosen 1% of Taiwan's population (about 200,000 individuals). The enrollment files contained individual subscription information and demographic factors, including sex, date of birth, type of beneficiaries and location. The claims files contained comprehensive records of inpatient care, ambulatory care, pharmacy store, dental care and Chinese medicine services, including date of service, ICD-9-CM (International Classification of Diseases) diagnosis codes, claimed medical expenses and the amount of co-payment for each encounter. The requirement was 12-months enrollment in year 2002 for concurrent analyses, while 24 months enrollment in years 2002 and 2003 were required for prospective analyses. The final sample size was 173,234 in the concurrent and 164,562 in prospective analyses.

Annual health expenditures were aggregated from all inpatient, outpatient and pharmacy store claimed expenses for every enrollee, including claimed reimbursement, medication expenses and co-payments; expenses for dental care and Chinese medicine were excluded from this aggregation. The total expenditure could be further divided into inpatient/outpatient/pharmacy store expenditure, or medical/drug/pharmacy service expenditure. Given that pharmacy store and pharmacy service expenditure was very small (each accounted for less than 2.5% of the total expenditure), results of both categories were not reported. The 2002 expenditure was used for concurrent analyses while year 2003 expenditure was used for prospective analyses. The unit of money in Taiwan is the New Taiwan Dollar (NTD); the exchange rate is about 32 NTD: 1 US dollar as of November 2009. Demographic factors included: sex; type of beneficiaries (insured or dependent); categorical age (0-17, 18-34, 35-49, 50-64, 65+); insurance category (based on insured's type of job); residence (three levels with different degrees of population density); and locality (six regions: Taipei, Northern, Central, Southern, Kao-Ping and Eastern). Diagnosis-based risk adjustment factors, including ACGs, ADGs and EDCs, were derived from the ACG case-mix system (Version 7.1) using the individuals' overall ICD-9-CM codes from both inpatient and outpatient records in 2002 (diagnosis codes from dental care and Chinese medicine were excluded).

### The ACG risk adjustment system

ACG actuarial cells are mutually exclusive health status categories defined by morbidity pattern, age and sex. The ACG system assigns all ICD-9-CM codes to one of 32 diagnostic clusters (ADGs) based on five clinical dimensions: duration; severity; diagnostic certainty; aetiology; and specialty care involvement [23,24]. Each ADG is a grouping of diagnosis codes similar in terms of severity and likelihood of persistence of the health condition treated over a relevant period of time, typically 1 year. ADGs are not mutually exclusive and individuals can have multiple ADGs (up to 32). Individuals are then placed into one of 93 discrete ACG categories according to their assigned ADGs, age and sex. The result is that individuals within a given ACG experienced a similar pattern of morbidity and resource consumption. The Johns Hopkins EDC methodology assigns each ICD code to a single disease category or EDC; there are 264 EDCs in total. ICD codes within an EDC share similar clinical characteristics and are expected to induce similar types of diagnostic and therapeutic responses.

### Measuring predictive performance

The following risk adjustment models (from the simplest to the most comprehensive) were used to explain five types of expenditure (total, inpatient, outpatient, medical and drug), both concurrently and prospectively:

1. Demographics only,

2. ACGs only,

3. ADGs with demographics,

4. ADGs plus selected EDCs with demographics, and

5. Full EDCs with demographics.

Selected EDCs were derived from the results of stepwise analyses using all EDCs (Additional file 1) and the final set of selected EDCs were different in concurrent (33 EDCs) and prospective analyses (19 EDCs). As expenditure is a non-negative variable, negative predicted expenditures from models were set at zero.

The performance of five risk adjustment models was evaluated at two levels: adjusted R^2 ^and mean absolute prediction error (MAPE) [22,36] at individual level, and predictive ratio (PR) at group level. MAPE was the average of all absolute differences between the observed and the predicted. MAPEs of different types of expenditure were divided by their respective means so that results on different types of expenditures could be compared. PR was calculated by dividing mean predicted expenditure by mean actual expenditure within a selected group of subjects. The model performed better if R^2 ^was larger, MAPE was smaller and the PR was closer to one. Split analysis was performed (a randomly selected 70% of study subjects were used for model development while the rest was set aside for model validation), and measures of model performance were obtained from the validation set to avoid overfitting.

Among these three indicators, R^2 ^was easily influenced by outliers [37]. Therefore, three models with different levels of truncation were performed in order to reduce the influence of outliers: no truncation (raw expenditure); truncation at two standard deviations above mean of log expenditure plus one; and truncation at the top 0.5%. Definitions of groups used to calculate PR included actual total expenditure quintiles, disease burden and age/sex group. Disease burden was classified into six categories from very low to very high morbidity and was also based on an output of the ACG system. In concurrent analyses, group classification could only be based on the 2002 (current) information; in prospective analyses, however, group classification could be based on either the 2002 (prior) or the 2003 (current) information.

### Statistical analysis

All statistical analyses were conducted using SAS™ software version 9.1. Several statistical methods had been proposed for the analysis of expenditure and no single method was seen to be the best under different conditions examined in these studies [38-40]. Comparisons of these statistical models for the explanation of expenditure are presented in Additional file 2. In this study, given the very high R^2^, the comparable MAPE, the standard approach usually adopted in studies involving risk adjustment [41,42] and a very large sample size [16], the ordinary least squares (OLS) regression model was used.

## Results

### Characteristics of the population (Table 1)

**Table 1:** Characteristics of the Taiwanese population for concurrent and prospective analyses

	Concurrent	Prospective
**Inclusion criteria**	**2002 12 month enrollment**	**2002 & 2003 24 month enrollment**

**No. of observations**	173,234	164,562

**Male**	50.16%	49.35%
**Insured**	40.77%	40.93%
**Age in 2002**	34.86	34.98
**Age group in 2002**		
0 - 17	23.29%	23.84%
18 - 34	28.22%	26.83%
35 - 49	24.96%	25.51%
50 - 64	13.83%	14.19%
65+	9.70%	9.62%
**Bureau of National Health Insurance branch**		
Taipei	32.68%	32.52%
North	14.84%	14.81%
Central	19.78%	19.94%
Southern	14.24%	14.42%
Kao-Pin	16.10%	16.03%
Eastern	2.32%	2.27%
**Residence level**		
Special municipality	21.66%	21.31%
City	14.94%	15.13%
County	63.37%	63.56%
**Medical utilization**	Year *2002*	Year *2003*
1+Outpatient visit/expenditure	89.99%	89.71%
1+Inpatient visit/expenditure	8.04%	7.17%
1+ Drug expenditure	89.46%	87.77%
1+ Total expenditure	90.38%	90.05%
		
Total expenditure (NTD/year)*	14,279	14,741
Medical expenditure (NTD/year)*	9,968	10,214
Pharmacy expenditure (NTD/year)*	3,912	4,108
Inpatient expenditure (NTD/year)*	4,781	4,788
Outpatient expenditure (NTD/year)*	9,245	9,577

1 US dollar = 32 New Taiwan dollar (NTD) as of November 2009

The distribution of demographic factors and medical utilization was similar among all subjects included in concurrent and prospective analyses. About half of the study subjects were male and 40% were the insured. The mean age in 2002 was 35 years and 10% were elderly. About one-third lived in the areas within the Taipei Branch, while only 2% were from the Eastern Branch. About 65% were living in rural county areas. Only 10% had not made any outpatient visit, while 8% had at least one inpatient stay. About 90% had non-zero total expenditure and a similar percentage had non-zero drug expenditure. The 2003 expenditure of the prospective sample were about the same as the 2002 expenditure of the concurrent sample. Mean total expenditure was about 14,500 NTDs, among which medical expenditure (10,000 NTDs) was much higher than pharmacy expenditure (4000 NTDs). Outpatient expenditure (9500 NTDs) was also much higher than inpatient expenditure (4800 NTDs).

### Proportion of total variance explained by the models (adjusted R^2^) (Tables 2 and 3)

**Table 2:** Concurrent adjusted R-squared and mean absolute prediction error of alternate risk factors and different categories of expenditure.

	Total expenditure	Type of expenditure		Source of expenditure	
		
		Medical	Pharmacy	Inpatient	Outpatient
**R^2^: (1) raw expenditure**					

Demographics-only	0.0421	0.0305	0.0266	0.0132	0.0477
ACGs	0.1486	0.1319	0.0597	0.0894	0.1107
ADGs and demographics	0.1709	0.1435	0.0848	0.0976	0.1342
ADGs, 33 EDCs and demographics	0.3895	0.3938	0.155	0.3053	0.3156
264 EDCs and demographics	0.3972	0.3993	0.1638	0.3191	0.3216

**R^2^: (2) expenditure truncated at 2 standard deviations above mean of log (expenditure+1)**					

Truncated criterion	595,431	339,360	86,013	262,810	449,657
% of truncated cases	0.24%	0.33%	0.32%	0.33%	0.15%

Demographics-only	0.0701	0.0545	0.1664	0.0299	0.0855
ACGs	0.2275	0.2265	0.3012	0.1753	0.1935
ADGs and demographics	0.2599	0.2523	0.3843	0.1919	0.2323
ADGs, 33 EDCs and demographics	0.4825	0.4492	0.4738	0.3643	0.4321
264 EDCs and demographics	0.4969	0.4695	0.5238	0.3994	0.4409

**R^2^: (3) expenditure truncated at top 0.5%**					

Truncated criterion	333,235	250,144	70,948	193,296	115,412
% of truncated cases	0.47%	0.48%	0.44%	0.51%	0.45%

Demographics-only	0.0963	0.0629	0.1785	0.0323	0.1836
ACGs	0.298	0.2576	0.3162	0.1845	0.402
ADGs and demographics	0.3419	0.2873	0.4032	0.2051	0.4767
ADGs, 33 EDCs and demographics	0.517	0.4618	0.4855	0.3626	0.5539
264 EDCs and demographics	0.5414	0.488	0.5377	0.4021	0.588

**Mean absolute prediction error: raw expenditure**					

Demographics-only	109.19%	118.23%	111.02%	174.62%	94.09%
ACGs	86.72%	94.69%	93.40%	144.80%	74.21%
ADGs and demographics	94.42%	105.85%	95.91%	164.70%	75.33%
ADGs, 33 EDCs and demographics	77.93%	86.17%	87.61%	133.71%	68.89%
264 EDCs and demographics	77.57%	87.51%	83.40%	133.32%	68.34%

Number in study sample (validation only): 51,970

**Table 3:** Prospective adjusted R^2 ^and mean absolute prediction error of alternate risk factors and different categories of expenditure.

	Total expenditure	Type of expenditure		Source of expenditure	
		
		Medical	Pharmacy	Inpatient	Outpatient
**R^2^: (1) raw expenditure**					

Demographics-only	0.0442	0.0308	0.0323	0.0146	0.0435
ACGs	0.0853	0.0643	0.0531	0.0312	0.0833
ADGs and demographics	0.1014	0.0714	0.0752	0.0353	0.1022
ADGs, 19 EDCs and demographics	0.2194	0.2154	0.1310	0.0797	0.3285
264 EDCs and demographics	0.2250	0.2196	0.1481	0.0842	0.3281

**R^2^: (2) expenditure truncated at 2 standard deviation above mean of log (raw expenditure+1)**					

Truncated criterion	618,385	344,754	98,239	294,026	462,867
% of truncated cases	0.27%	0.37%	0.32%	0.29%	0.19%

Demographics-only	0.0635	0.0516	0.1489	0.0281	0.0710
ACGs	0.1187	0.1015	0.2202	0.0562	0.1305
ADGs and demographics	0.1427	0.1179	0.2943	0.0661	0.1594
ADGs, 19 EDCs and demographics	0.2746	0.2130	0.3483	0.0902	0.3670
264 EDCs and demographics	0.2830	0.2193	0.3972	0.0989	0.3740

**R^2^: (3) expenditure truncated at top 0.5%**					

Truncated criterion	356,940	276,041	74,088	194,860	124,309
% of truncated cases	0.49%	0.48%	0.47%	0.50%	0.48%

Demographics-only	0.0889	0.0579	0.1662	0.0326	0.1664
ACGs	0.1580	0.1126	0.2427	0.0611	0.2819
ADGs and demographics	0.1920	0.1310	0.3227	0.0728	0.3454
ADGs, 19 EDCs and demographics	0.2814	0.2105	0.3814	0.0935	0.4207
264 EDCs and demographics	0.2960	0.2177	0.4283	0.1031	0.4495

**Mean absolute prediction error: raw expenditure**					

Demographics-only	111.76%	120.43%	115.22%	180.28%	97.42%
ACGs	102.21%	112.01%	105.59%	172.66%	87.81%
ADGs and demographics	103.64%	115.56%	104.12%	175.53%	87.85%
ADGs, 19 EDCs and demographics	95.83%	106.84%	97.97%	170.66%	80.84%
264 EDCs and demographics	96.52%	108.51%	97.30%	172.85%	81.81%

Number of study sample (validation only): 49,369.

In concurrent analyses, the demographic model explained 4%, the ACGs and ADGs models explained about 15%, the ADGs plus selected EDCs model and full EDCs model explained roughly 40% of variances in the total expenditure. In prospective analyses, the demographic model explained 4%, the ACGs and ADGs models explained about 10%, the ADGs plus selected EDCs model and full EDCs model explained over 20% of variances. In concurrent analyses, the adjusted R^2 ^of medical expenditures was much higher than pharmacy expenditure across all models. In the prospective analyses, the adjusted R^2 ^of medical expenditure was only higher among the two EDCs-related models while comparable in other models. In addition, in concurrent analyses, the adjusted R^2 ^of outpatient expenditures was slightly higher in simpler models while comparable to that of inpatient expenditure in the two EDC-related models. In prospective analyses, the adjusted R^2 ^of outpatient expenditure was always higher across all models. Comparing adjusted R^2 ^of concurrent and prospective analyses, it was found that the lower adjusted R^2 ^of total expenditure in prospective analyses was the result of the lower prospective adjusted R^2 ^of medical and inpatient expenditure (the adjusted R^2 ^of pharmacy and outpatient expenditure from both concurrent and prospective analyses was similar).

In both concurrent and prospective analyses, truncation increased adjusted R^2 ^across all types of expenditure, especially in pharmacy and outpatient expenditure. The only exception was that the adjusted R^2 ^of prospective medical expenditure in the EDC-related models remained the same after truncation. After truncation, the adjusted R^2 ^was higher in pharmacy expenditure (compared to medical expenditure) and outpatient expenditure (relative to inpatient expenditure), and the differences of adjusted R^2 ^(pharmacy/medical expenditure and inpatient/outpatient expenditure) were much larger in prospective analyses. We also found that the adjusted R^2 ^of pharmacy expenditure increased the most after truncation. In addition, it also showed that the more the number of observations truncated, the higher the adjusted R^2^. After truncation at the top 0.5%, the adjusted R^2 ^of total expenditure in two EDCs models, increased from 40% to 53% in concurrent analyses and from 22% to 29% in prospective analyses; such an increase was larger in concurrent analyses.

Adjusted R^2 ^was different between the elderly and non-elderly group (Table 4). In concurrent analyses, the adjusted R^2 ^in four ACG-related risk adjustment models was always larger in the elderly population, with the exception of the adjusted R^2 ^of outpatient expenditure from the two EDCs models. The biggest difference was the adjusted R^2 ^of inpatient expenditure. It was about 20 percentile larger in the elderly population in the EDC-related models. The adjusted R^2 ^of total expenditure in the non-elderly population ranged from 1.4% in the demographic model to 33% in the EDCs-related models, while in the elderly population it was from 0.4% to 45%. In the prospective analyses, the adjusted R^2 ^in the elder population was only larger in pharmacy expenditure, while smaller or similar in all other expenditure. The adjusted R^2 ^in the non-elderly population ranged from 1.7% in the demographic model to 22% in the EDC-related models, while in the elderly population it was from 0.3% to 16%. Demographic models performed badly in the elderly population in both prospective and concurrent analyses.

**Table 4:** Concurrent and prospective adjusted R-Squared of raw expenditures by two age groups.

	Total expenditure	Type of expenditure		Source of expenditure	
		
		Medical	Pharmacy	Inpatient	Outpatient
**Concurrent analyses**					

**The non-elderly population (*N *= 46,895)**					
Demographics-only	0.014	0.012	0.006	0.004	0.018
ACGs	0.105	0.1	0.032	0.06	0.076
ADGs and demographics	0.133	0.12	0.05	0.079	0.094
ADGs, 33 EDCs and demographics	0.325	0.348	0.105	0.211	0.316
264 EDCs and demographics	0.329	0.355	0.105	0.219	0.324

**The elderly population (*N *= 5,075)**					

Demographics-only	0.004	0.001	0.013	0.002	0.005
ACGs	0.158	0.133	0.114	0.113	0.105
ADGs and demographics	0.197	0.164	0.165	0.149	0.137
ADGs, 33 EDCs and demographics	0.452	0.433	0.233	0.418	0.279
264 EDCs and demographics	0.445	0.425	0.229	0.421	0.273

**Prospective analyses**					

**The non-elderly population (*N *= 44,628)**					

Demographics-only	0.017	0.014	0.008	0.005	0.018
ACGs	0.059	0.049	0.025	0.023	0.055
ADGs and demographics	0.072	0.056	0.038	0.026	0.068
ADGs, 19 EDCs and demographics	0.215	0.237	0.112	0.04	0.351
264 EDCs and demographics	0.218	0.242	0.124	0.045	0.35

**The elderly population (*N *= 4,741)**					

Demographics-only	0.003	0.002	0.009	0.002	0.003
ACGs	0.063	0.042	0.115	0.023	0.074
ADGs and demographics	0.075	0.047	0.164	0.03	0.096
ADGs, 19 EDCs and demographics	0.166	0.155	0.178	0.097	0.263
264 EDCs and demographics	0.155	0.14	0.196	0.094	0.248

### Mean absolute prediction error (%) (Tables 2 and 3)

In concurrent analyses, MAPE of total expenditure in the demographic model was 109%; that from the ACG model was 87%, which was better than the ADGs model (94%), and the MAPEs from the two EDC-related models were roughly the same (78%). In the prospective analyses, the MAPE of total expenditure in the demographic model was 112%. Those of the ACG and ADG models were close (103%), while the MAPEs of the two EDCs-related models were about 96%. Both concurrent and prospective analyses showed that MAPEs were the smallest in outpatient expenditures, then total, pharmacy, and medical expenditures, while the largest MAPEs were from inpatient expenditures. MAPEs of outpatient expenditures were about half of those from inpatient expenditures across all models. In addition, MAPEs were smaller in concurrent analyses than in prospective analyses.

### Predictive ratio (PR) (Tables 5 and 6)

**Table 5:** Concurrent predictive ratios of alternate risk factors and different categories of expenditures.

Group	Mean expenditure	Demographics	ACGs	ADGs and demographics	ADGs, 33 EDCs and demographics	264 EDCs and demographics
**Expenditure group by quintiles (current classification: based on 2002)**						

0 - 20	267.1	44.127	5.384	6.667	6.034	7.587
20 - 40	1634.4	6.316	3.282	2.845	2.362	2.197
40 - 60	3739.1	2.941	2.439	2.359	1.92	1.742
60 - 80	2898.3	1.658	1.963	2.026	1.556	1.445
80 -100	57344	0.433	0.687	0.752	0.81	0.828

**Morbidity burden (current classification: based on 2002 morbidity level)**						

Non-user	7.6	1688.88	0.924	186.889	170.046	254.982
Very low	1750.8	5.511	1.138	0.407	0.645	0.935
Low	4282	2.323	0.931	0.924	0.932	1.011
Moderate	13290.6	1.168	1.044	1.203	1.07	1.051
High	38526.2	0.561	0.948	1.108	0.991	0.954
Very High	106731.5	0.313	1.028	0.861	0.971	0.955

**Demographic group (sex and age group)**						

M, 0~18	6312.9	1.982	1.01	1.077	0.981	1.043
M, 18~34	7037.6	1.9	0.949	1.097	1.016	0.997
M, 35~49	10971.9	1.344	1.135	1.058	1.006	0.976
M, 50~64	22590.3	0.679	0.928	0.911	0.975	0.973
M, 65+	50066.9	0.381	0.798	0.887	0.992	1.023
F, 0~18	6989	1.8	0.887	0.932	0.843	0.89
F, 18~34	8221.8	1.676	1.178	1.388	1.167	1.053
F, 35~49	11974.7	1.208	1.224	1.208	0.993	0.955
F, 50~64	22404.3	0.689	1.137	1.141	1.016	1.009
F, 65+	43574.6	0.384	0.931	0.974	1.031	1.052
						

**Table 6:** Prospective predictive ratios of alternate risk factors and different categories of expenditures.

Group	Mean expenditure	Demographics	ACGs	ADGs and demographics	ADGs, 19 EDCs and demographics	264 EDCs and demographics
**Expenditure group by quintiles (prior classification: based on 2002)**						

0 - 20	276.2	3.915	1.427	1.359	1.374	1.617
20 - 40	1646.6	2.212	1.5	1.222	1.189	1.177
40 - 60	3737.7	1.573	1.455	1.3	1.181	1.122
60 - 80	8270	1.31	1.582	1.5	1.318	1.246
80 -100	51801.4	0.537	0.727	0.811	0.872	0.888

**Expenditure group by quintiles (current classification: based on 2003)**						

0 - 20	233.2	52.474	29.365	28.362	27.018	28.393
20 - 40	1513.5	6.949	5.456	4.68	4.474	4.507
40 - 60	3571.8	3.187	3.093	2.76	2.492	2.424
60 - 80	8192.6	1.736	1.982	1.889	1.679	1.632
80 -100	59354.1	0.43	0.525	0.587	0.641	0.649

**Morbidity burden (prior classification: based on 2002 morbidity level)**						

Non-user	3013.8	4.432	1.216	1.449	1.411	1.754
Very low	4081.3	2.483	1.046	0.636	0.837	1.032
Low	5746.9	1.775	0.95	0.879	0.968	1.022
Moderate	15844.5	1.019	1.002	1.05	1.003	0.983
High	34475.8	0.64	1.019	1.099	1.061	1.026
Very high	78192.9	0.429	1.012	0.922	0.984	0.985

**Morbidity burden (current classification: based on 2003 morbidity level)**						

Non-user	8.2	1592.85	726.045	794.094	755.867	811.262
Very low	1651	6.124	4.273	3.597	3.473	3.641
Low	3965.4	2.571	2.143	1.87	1.798	1.826
Moderate	14477.2	1.125	1.165	1.183	1.168	1.152
High	38039.1	0.578	0.76	0.812	0.808	0.802
Very high	110996	0.301	0.439	0.48	0.529	0.539

**Demographic group (sex and age group)**						

M, 0~18	6932	1.815	1.005	1.075	1.065	1.097
M, 18~34	7654	1.841	1.018	1.082	1.012	1.041
M, 35~49	11403	1.35	1.218	1.069	1.077	1.059
M, 50~64	24776.7	0.642	0.905	0.835	0.863	0.859
M, 65+	51787.1	0.386	0.672	0.761	0.85	0.901
F, 0~18	4976.6	2.554	1.38	1.478	1.401	1.409
F, 18~34	7948.9	1.799	1.228	1.392	1.252	1.171
F, 35~49	12074.5	1.253	1.348	1.223	1.125	1.074
F, 50~64	24265.9	0.664	1.054	0.998	1.003	0.973
F, 65+	43298.1	0.401	0.846	0.882	0.95	1.009

#### Expenditure levels by quintiles

All models underpredicted total expenditure in the highest quintile group while expenditure was overpredicted in the four other groups. When groups were defined based on current information (2002 expenditure for the concurrent and 2003 expenditure for the prospective analyses), PR decreased from the lowest to the highest quintile group. There was an especially large drop moving from the lowest to the second lowest group. Among the current classification, PR was smaller in concurrent than prospective analyses. When groups were defined using prior classification (in prospective analyses only), the decreasing trend was not clear and PR was much smaller. In general, comprehensive models usually performed better than simpler models and the demographic model performed much worse than the other models.

#### Morbidity status

Overall, comprehensive models tended to perform better with the exception of the ACG model. The ACG model performed far better than all other models in concurrent analyses. PR of people with the lowest morbidity burden was only about 0.9 in the ACG model but was more than 100 in the other models. Similarly, all models tended to overpredict the total expenditure for people with the lower morbidity burden, who had lower total expenditure, while underpredicted expenditure for people with higher morbidity burden (hence higher total expenditure). There was no decreasing trend for PR other than that in the demographic model or current classification in prospective analyses. PR based on current classification usually deviated further from 1 in prospective than in concurrent analyses. PR based on prior classification was much better than that on current classification, probably because the difference in mean expenditures across groups was much smaller.

#### Age/sex group

Comprehensive models tended to perform better. Among younger groups, PR deviated further from 1 in females. However, among elder groups, on the contrary, deviation was larger in males. Overall, PR was closer to 1 in male compared to the female groups in both concurrent and prospective analyses. In addition, there was a tendency for all models to overpredict total expenditure in the elder groups while underpredicting in the younger groups in both genders, especially in prospective analyses and simpler models.

## Discussion

We found that the adjusted R^2 ^of total expenditures in concurrent/prospective analyses was about 4% in the demographic model, 15%/10% in the ACGs or ADGs models and 40%/22% in the models containing EDCs. The adjusted R^2 ^of medical/outpatient expenditure was always larger than that of pharmacy/inpatient expenditure. The performance of the ADGs plus selected EDCs models was comparable to that of the full EDCs model. When predicting expenditure for groups based on expenditure quintiles, all models underpredicted the highest group while overpredicting the other four groups. For population sub-groups selected on morbidity burden, however, the ACGs model had the best performance overall.

The prerequisite for adopting diagnosis-based risk adjustment models is that individuals' diagnosis information has to be complete and available. Given the consistently high enrollment rate (99% by the end of 2006 [43]), the high NHI-contracted rate of providers (above 90% [43,44]), a comprehensive benefit package and the centralization of claims data, diagnosis information should be able to capture an individual's morbidity information and is readily available in Taiwan. Among the 1.25 million unique diagnoses encountered by 173,234 subjects, only 0.393% were non-grouped and 0.78% were unknown to the ACG system. Given the very small number in both cases, it provided face validity in the quality of diagnosis and the ability of the ACG system to process claims data in Taiwan.

One possible weakness of the study is that the coding may have improved over the study years so that people with the same condition had more complete ICD codes reported if they used medical services during a latter period. Therefore, we examined the number of ICD codes reported for each person from 2000 to 2003. If the number did not differ very much, it would imply that the improved coding might not be a problem for this analysis. The numbers of ICD codes assigned to each patient from year 2000 to 2003 were: 17.86, 18.07, 18.62 and 18.20, respectively. Given this slight variation, it seemed that the increased coding was not likely to be a problem for this study.

Several risk adjusters have been evaluated in Taiwan, including catastrophic disease status [45], prior utilization [45-48], diagnosis-based models [45-47,49,50] and pharmacy-based models [34,51]. It was found that prior utilization yielded the highest R^2^. Diagnosis-based models performed better than pharmacy-based models, while the catastrophic disease status was somewhat less efficient than the pharmacy-based models. The ACG system has been examined in several studies [35,49,50,52]. Given the difference in the truncation levels, statistical methods and how expenditure was calculated, it was difficult to make direct comparisons. However, the general findings that the ACGs/ADGs categorical model did not perform as well as other claims-based risk adjustment models that document individual diseases (such as the EDCs) and that the adjusted R^2 ^of outpatient expenditure was much higher than that of inpatient expenditure, still hold in this study.

The performance of the ACGs/ADGs model in Taiwan was comparable to what the models had achieved among the general population in other countries. This in part suggested that the ACG system can be directly applied to Taiwan's NHI system. The lower R^2 ^performance of both categorical models compared to other disease-specific diagnosis-based models is probably due to the limited numbers of variables included in both models and the difference in the grouping algorithm (ACGs has 93 mutually exclusive categories and ADGs consist of only 32 binary variables). However, after adding selected disease indicators to the ADGs model, the performance was comparable to what could be achieved by other diagnosis-based models (40% concurrently and 22% prospectively in raw total expenditure). This finding was consistent with results from previous research that patients of some common and expensive chronic diseases accounted for a relatively large proportion of healthcare costs and adding these disease indicators improved the predictive power of the risk adjustment models [15,17]. It may be necessary to incorporate important disease indicators if the ACG system were used. That is the approach used by the current ACG-PM model in ACG version 7.0 and after.

Quality improvement and financial balance are two of three main goals of Taiwan's NHI reform set up by the NHI's Second Generation Planning Committee [33] and both require strong risk adjustment tools. One major approach suggested by the Committee to achieve quality improvement is to release valid and understandable quality information regarding healthcare providers to the public in order that beneficiaries can make informed decisions. However, before quality information can be released, it is important and necessary to implement risk adjustment so that patient differences across healthcare organizations are controlled for and variation in the quality of care can be attributed to providers. In addition, the Planning Committee also concluded that the payment system reform should involve healthcare providers in taking on more financial responsibility for containing costs and it was suggested that the per-case payments and partial capitation should replace the current fee-for-service payment system [33]. The implementation of risk adjustment is necessary in order to ensure equity if any form of capitation or budgeted payment system is used in the future. Both of these issues are likely to be applicable to most other developed healthcare systems around the globe.

Given the availability and comprehensiveness of claims data, Taiwan has the necessary information to implement diagnosis-based risk adjustment. This study further shows that the ACG case-mix system performs comparably to diagnosis-based risk adjustment models applied in other health care systems and far better than the demographics-only model currently employed for the NHI. Therefore, incorporating diagnosis-based risk adjustment into NHI will be a major task facing healthcare policymakers and administrators in Taiwan.

## Limitations

The ACG system was developed using American health insurance claims; given the differences in healthcare systems and care-seeking behaviours, it may be necessary to adjust the risk classification system inherent in the model so that it can reflect the local patterns of disease burden and health services utilization, such as Chinese medicine.

The calculation of an individual's enrollment period was a concern in this study. As only an individual's latest enrollment record was included in the yearly enrollment files starting from 2003, it was only possible to calculate the exact length of enrollment before 2003 but not afterwards. It was assumed in this study that all subjects in the 2003 file were enrolled in NHI starting from January 2003 for the following reasons: (1) the enrollment type of all enrollment records in 2003 was the same - 'transferring in' indicates that an individual had a new enrollment record because of the change in insurance identity or unit and they were all enrolled prior to this change; (2) the enrollment rate was consistently high in Taiwan; (3) the distribution of individuals' length of enrollment in 2003 and after, based on this assumption, was similar to that in 2002 or earlier. The effect of this assumption was that some subjects who were not 12-month enrollees in 2003 would be included in the study.

In this analysis people who did not have continuous enrollment over the study period were excluded. This led to some differences of characteristics between subjects in the analysis sample and the target population, which may have moderately affected the generalizability of the results. It was found that there were statistically significantly differences in demographics and medical utilization between those who had full 12-month enrollment and those who did not in 2002 (Table 7) and between those having 24-month enrollment in both 2002 and 2003 and those having 12-month enrollment only in 2002 (Table 8). Those excluded from the analyses had a much higher average healthcare expenditure and inpatient visits (even though they did not have full-year enrollment), although a higher proportion of them did not use any medical service.

**Table 7:** Characteristics of subjects with continuous and incomplete enrollment among 2002 enrollees (*N *= 181,790).

	2002 1~11 month enrollment	2002 12 month enrollment
**Number of observations**	**8,556**	**173,234**

**Male**	56.42%	50.16%
**Insured**	30.89%	40.77%
**Age in 2002**	32.38	34.86
**Age group in 2002**		
0 - 17	15.45%	23.29%
18 - 34	50.41%	28.22%
35 - 49	18.70%	24.96%
50 - 64	7.47%	13.83%
65+	7.97%	9.70%
**Bureau of National Health Insurance Branch in 2002**		
Taipei	39.96%	32.68%
North	16.78%	14.84%
Central	16.66%	19.78%
Southern	11.00%	14.24%
Kao-Pin	13.35%	16.10%
Eastern	2.25%	2.32%
**Residence level in 2002**		
Special municipality	26.57%	21.66%
City	14.56%	14.94%
County	58.87%	63.37%
Medical utilization in 2002*		
1+ Outpatient visit/expenditure	76.26%	89.99%
1+ Inpatient visit/expenditure	11.76%	8.04%
1+ Drug expenditure	76.34%	89.46%
1+ Total expenditure	77.57%	90.38%
		
Total expenditure (NTD/year)†	23,351	14,279
Medical expenditure (NTD/year) †	17,677	9,968
Pharmacy expenditure (NTD/year) †	5,309	3,912
Inpatient expenditure (NTD/year) †	17,209	4,781
Outpatient expenditure (NTD/year) †	6,007	9,245

* For those without 12 full months enrollment, the actual utilization is underestimated.

†1 US dollar = 32 New Taiwan dollar (NTD) as of November 2009

**Table 8:** Characteristics of subjects with continuous and incomplete enrollment in 2003 among 2002 continuous enrollees.

	2002 12 month enrollment only	2002 and 2003 24 month enrollment only
**Number of observations**	**8672**	**164,562**

**Male**	65.59%	49.35%
**Insured**	37.80%	40.93%
**Age in 2002**	32.73	34.98
**Age group in 2002**		
0 - 17	12.88%	23.84%
18 - 34	54.60%	26.83%
35 - 49	14.56%	25.51%
50 - 64	6.85%	14.19%
65+	11.10%	9.62%
**Bureau of National Health Insurance Branch in 2002**		
Taipei	34.79%	32.59%
North	15.77%	14.80%
Central	17.60%	19.90%
Southern	11.55%	14.39%
Kao-Pin	17.44%	16.04%
Eastern	2.85%	2.29%
**Residence level in 2002**		
Special municipality	26.09%	21.43%
City	14.03%	14.99%
County	59.88%	63.58%
**Medical utilization in 2002**		
1+ Outpatient visit/expenditure	83.38%	90.34%
1+ Inpatient visit/expenditure	14.00%	7.73%
1+ Drug expenditure	83.15%	89.79%
1+ Total expenditure	83.99%	90.72%
		
Total expenditure (NTD/year)*	31,962	13,348
Medical expenditure (NTD/year)*	23,835	9,238
Pharmacy expenditure (NTD/year)*	7,586	3,718
Inpatient expenditure (NTD/year)*	20,141	3,972
Outpatient expenditure (NTD/year)*	11,640	9,119

*1 US dollar = 32 New Taiwan dollar (NTD) as of November 2009

The reason for this seemingly conflicting result might be that the group without full enrollment mainly consisted of two different types of people: those who died during the year and those who served in the army in that year (and thus were removed from this dataset for national security reasons). People tended to consume a lot more medical resources before they died, so the average expenditure would increase hugely. On the other hand, those who served in the army were mostly in their twenties and, hence, less likely to use medical services and therefore the proportion of people using any service decreased. Therefore, the results of this study may not be fully generalizable to those who died during the year or were or may be in the army.

## Future research directions

Taiwan's NHI provides beneficiaries' comprehensive drug coverage. With drug information readily available, it will be interesting to evaluate how a pharmacy-based risk adjustment model, such as the ACG system's pharmacy-based morbidity groups (Rx-MG) measures, works in Taiwan and how much improvement can be made by including pharmacy information in the claims-based risk adjustment model. Furthermore, most diagnosis information used for risk adjustment models is cross-sectional, due in part to the difficulty of obtaining an individual's longitudinal diagnosis information. Given the universal and lifelong coverage under NHI in Taiwan, this setting provides a very good opportunity to examine how bringing in longitudinal claims data will affect the performance of risk adjustment models.

## Conclusions

Given the availability of claims data and the much better performance of claims-based risk adjustment models over the demographics-only model, Taiwan's government should incorporate claims-based models in the important policy-setting processes, such as resource allocation, predictive modeling for high-risk case finding and cost prediction. The performance of the ACG risk adjustment system in Taiwan is comparable to that found in other countries; therefore, this suggests that the ACG system may be directly applied to Taiwan's NHI even though it was originally developed using USA claims data. In addition, it may be necessary to utilize the disease indicators component (EDCs) of the ACG system in order to ensure the highest performance of the ACG system. Given the experience in Taiwan, it is very likely that other nations will be able to apply the ACG system or other similar diagnosis-based risk adjustment tools if insurance claims or other computerized data sources capturing ambulatory and inpatient medical diagnoses are available.

## Abbreviations

ACG: Adjusted Clinical Group; ADG: Aggregated Diagnosis Group; BNHI: Bureau of NHI; EDC: Expanded Diagnosis Cluster; ICD: International Classification of Diseases; MAPE: mean absolute prediction error; OLS: ordinary least squares; NHI: National Health Insurance; NTD: New Taiwan dollar; PM: predictive modelling; PR: predictive ratio.

## Competing interests

HYC worked as a part-time research assistant for Dr Jonathan Weiner, one of the founders of the ACG system, when pursuing his PhD at Johns Hopkins University. The author is currently hired as post-doctorate fellow at John Hopkins University with part of the funding coming from the ACG team. JW is one of the developers of the ACG system. The Johns Hopkins University receives royalties for non-academic use of software based on the ACG methodology.

## Authors' contributions

HYC designed the study, cleaned the data, performed the statistical analysis and drafted the manuscript. JW provided insight into the concept and design of the study and revised the manuscript critically for important intellectual content. All authors read and approved the final manuscript.

## Pre-publication history

The pre-publication history for this paper can be accessed here:

http://www.biomedcentral.com/1741-7015/8/7/prepub

## Supplementary Material

Additional File 1 **Process of Generating Selected EDCs. **Appendix Click here for file

Additional File 2 **Comparison of Different Statistical Models. **Appendix Click here for file
