# Supplementary material for: An in-depth assessment of a diagnosis-based risk adjustment model based on national health insurance claims: the application of the Johns Hopkins Adjusted Clinical Group case-mix system in Taiwan
Source: BMC Med. 2010 Jan 18;8:7. doi: 10.1186/1741-7015-8-7 (PMC2830174; doi:10.1186/1741-7015-8-7)
Supplement: Additional File 1 — Process of Generating Selected EDCs. Appendix [file 1741-7015-8-7-S1.DOC]

Process of Generating Selected EDCs :

1. Demographic factors were forced into the model and EDCs entered the model with stepwise selection (significance level for entering in and removing from the model is 0.01).
2. Five types of expenditures (total amount, medical amount, drug amount, inpatient amount, outpatient amount) were used as dependent variables.
3. For each expenditure outcome, three types of age variables were tested (linear, categorical, and linear splines); therefore, for each expenditure variable, three models were constructed.
4. EDC variables with partial R^2^ greater than or equal to 0.001 were included for further consideration.
5. For each expenditure variable, only EDC variables included in at least two models (out of three different age models) were included for final consideration. Those EDCs on this final list were considered as ‘important’ for this specific expenditure variable.
6. Each EDC variable could be considered ‘important’ at any of five expenditure variables; below was the distribution of number of EDC variables by counts considered ‘important’ category.

| Counts considered ‘important’ in expenditures | 5 | 4 | 3 | 2 | 1 |
| --- | --- | --- | --- | --- | --- |
| Concurrent Analyses | | | | | |
| Number of EDC variables | 6 | 7 | 21 | 13 | 14 |
| Number of EDCs with observations <20 | 0 | 1 | 0 | 0 | 0 |
| Prospective Analyses | | | | | |
| Number of EDC variables | 4 | 2 | 15 | 6 | 9 |
| Cumulative number of EDCs variables | 0 | 0 | 2 | 0 | 2 |

1. EDCs with less than 20 observations were excluded to ensure the stability of the estimate. Only one EDC (New04, prematurity) satisfied this exclusion criterion (n=14) in concurrent analyses; in prospective analyses, four EDCs (NEW04-prematurity, MAL16-Acute Leukemia, INF04-HIV/AIDS, and GUR03-Hypospadias/Other Penile Anomalies) satisfied this exclusion criterion (n=13, 18, 2 and 10, respectively).
2. R^2^ of five models were calculated separately for concurrent and prospective analyses.

|  | Model 1 | Model 2 | Model 3 | Model 4 | Model 5 |
| --- | --- | --- | --- | --- | --- |
| Demographics | √ | √ | √ | √ | √ |
| EDCs with counts of importance | 5 | 4, 5 | 3~5 | 2~5 | 1~5 |
| R2 of concurrent total expenditures | -- | 35.2% | 39.4% | 40.4% | -- |
| R2 of prospective total expenditures | -- | 17.3% | 18.5% | 18.6% | 18.9% |

1. There was a large drop of R^2^ from Model 3 to Model 2 in both concurrent and prospective analyses. Therefore, EDCs included in Model 3 were chosen.

**List of Conditions**

| Selected Conditions | Concurrent | Prospective |
| --- | --- | --- |
| Acute Myocardial Infarction | √ |  |
| Adverse Events from Medical/Surgical Procedures | √ |  |
| Cardiac Arrest, Shock | √ | √ |
| Cardiovascular Disorders, Other |  | √ |
| Central Nervous System Infections | √ | √ |
| Cerebral Palsy | √ |  |
| Cerebrovascular Disease | √ | √ |
| Chronic Renal Failure | √ | √ |
| Chronic Ulcer of the Skin | √ |  |
| Complications of Mechanical Devices | √ | √ |
| Congestive Heart Failure | √ |  |
| Deep Vein Thrombosis | √ |  |
| Depression | √ |  |
| Diabetic Retinopathy |  | √ |
| Disorders of the Immune System | √ |  |
| Gastrointestinal/Hepatic Disorders, Other | √ |  |
| Hemophilia, Coagulation Disorder | √ | √ |
| High Impact Malignant Neoplasms | √ | √ |
| Hypertension, w/o Major Complications |  | √ |
| Ischemic Heart Disease (excl Acute Myocardial Infarction) | √ | √ |
| Malignant Neoplasms, Colorectal | √ | √ |
| Malignant Neoplasms, Kidney | √ |  |
| Malignant Neoplasms, Liver and Biliary Tract | √ | √ |
| Malignant Neoplasms, Prostate | √ |  |
| Nephritis, Nephrosis |  | √ |
| Paralytic Syndromes, Other | √ |  |
| Peripheral Vascular Disease | √ |  |
| Respiratory Disorders, Other | √ |  |
| Respiratory Failure | √ | √ |
| Schizophrenia and Affective Psychosis | √ | √ |
| Seizure Disorder | √ |  |
| Septicemia | √ | √ |
| Spinal Cord Injury/Disorders | √ |  |
| Surgical Aftercare | √ |  |
| Tracheostomy | √ | √ |
| Transplant Status | √ |  |
| Type 2 Diabetes, w/o Complication | √ | √ |
| Total Number of Conditions | 33 | 19 |
