# Supplementary material for: An in-depth assessment of a diagnosis-based risk adjustment model based on national health insurance claims: the application of the Johns Hopkins Adjusted Clinical Group case-mix system in Taiwan
Source: BMC Med. 2010 Jan 18;8:7. doi: 10.1186/1741-7015-8-7 (PMC2830174; doi:10.1186/1741-7015-8-7)
Supplement: Additional File 2 — Comparison of Different Statistical Models. Appendix [file 1741-7015-8-7-S2.DOCX]

Comparison of Different Statistical Models

Several statistical methods had been proposed to analyze expenditures, and no single method was the best under different conditions examined in these studies. In this study, seven statistical methods were applied, including the two-part method, generalized linear model, and log-normal method, using all five sets of independent variables. Judging from adjusted R^2^, the OLS model consistently produced the highest R^2^ across all five sets of independent variables, both concurrently and prospectively. In terms of MAPE, even though the OLS model did not generate the lowest MAPE, MAPE from the OLS model was similar to what the best statistical method under consideration could achieve. Given the very high R^2^, comparable MAPE, the standard approach usually adopted in studies involving risk adjustment, and a very large sample size, the OLS regression model was employed in the study.

Adjusted R-squared and MAPE of concurrent & prospective total expenditure by different methods

|  | | **Reported method** | **Measure** | | **Best Method** | | **Best Measure** | |  |
| --- | --- | --- | --- | --- | --- | --- | --- | --- | --- |
| **Concurrent Analysis** (N=51,970; validation set) | | | | | | | | |  |
| **R-Squared** | | | | | | | | |  |
| Demographics | Ordinary Least Squares | | 0.0417 | | One-part GLM Poisson  One-part GLM Gamma  Two-part GLM Gamma | | 0.0424 | |  |
| ACGs | Ordinary Least Squares | | 0.1472 | | Ordinary Least Squares  One-part GLM Poisson  One-part GLM Gamma | | 0.1472 | |  |
| ADGs & Demographics | Ordinary Least Squares | | 0.1701 | | Ordinary Least Squares | | 0.1701 | |  |
| ADGs, 33 EDCs & Demographics | Ordinary Least Squares | | 0.3885 | | Ordinary Least Squares | | 0.3885 | |  |
| 264 EDCs & Demographics | Ordinary Least Squares | | 0.3939 | | Ordinary Least Squares | | 0.3939 | |  |
| **Mean Absolute Prediction Error** | | | | | | | | |  |
| Demographics | | Ordinary Least Squares | 109.19% | | Two-Part Lognormal – Normal Retransformation | | 108.78% | |  |
| ACGs | | Ordinary Least Squares | 86.72% | | Two-Part Lognormal – Smearing | | 81.03% | |  |
| ADGs & Demographics | | Ordinary Least Squares | 94.42% | | Two-Part Linear | | 90.12% | |  |
| ADGs, 33 EDCs & Demographics | | Ordinary Least Squares | 77.93% | | Two-Part Linear | | 75.67% | |  |
| 264 EDCs & Demographics | | Ordinary Least Squares | 77.57% | | Two-Part Linear | | 75.90% | |  |
| **Prospective Analysis** (N=49,369; validation set) | | | | | | | | | |
| R-Squared | | | | | | | | | |
| Demographics | | Ordinary Least Squares | | 0.0438 | | Ordinary Least Squares | | 0.0438 | |
| ACGs | | Ordinary Least Squares | | 0.0838 | | Ordinary Least Squares | | 0.0838 | |
| ADGs & Demographics | | Ordinary Least Squares | | 0.1004 | | Two-Part Linear | | 0.1008 | |
| ADGs, 19 EDCs & Demographics | | Ordinary Least Squares | | 0.2183 | | Ordinary Least Squares | | 0.2183 | |
| 264 EDCs & Demographics | | Ordinary Least Squares | | 0.2205 | | Ordinary Least Squares | | 0.2205 | |
| **Mean Absolute Prediction Error** | | | | | | | | | |
| Demographics | | Ordinary Least Squares | | 111.76% | | Two-Part Lognormal – Smearing | | 110.35% | |
| ACGs | | Ordinary Least Squares | | 102.21% | | Two-Part GLM Gamma | | 102.15% | |
| ADGs & Demographics | | Ordinary Least Squares | | 103.64% | | Two-Part GLM Poisson | | 99.22% | |
| ADGs, 19 EDCs & Demographics | | Ordinary Least Squares | | 95.83% | | Two-Part GLM Poisson | | 95.25% | |
| 264 EDCs & Demographics | | Ordinary Least Squares | | 96.52% | | Ordinary Least Squares | | 96.52% | |

Seven Statistical Methods Examined: ordinary least squares, two-part linear, one-part GLM with Gamma distribution, two-part GLM with Gamma distribution, one-part GLM with Poisson distribution, two-part lognormal with normal retransformation, two-part lognormal with Smearing retransformation.
